# P-1055. Clean Flush: Biological Validation of a Toilet-Based Human Waste Decontamination Protocol

**DOI:** 10.1093/ofid/ofaf695.1250

**Published:** 2026-01-11

**Authors:** Jessica Carag, Jill Morgan, Sarah Lohsen, Grace Drew, Dalia Gulick, Sarah W Satola, Colleen S Kraft

**Affiliations:** Emory University School of Medicine, Atlanta, Georgia; Emory University Hospital, Atlanta, Georgia; Emory University School of Medicine, Atlanta, Georgia; Emory University, Atlanta, GA; Emory University, Atlanta, GA; Emory University School of Medicine, Division of Infectious Diseases, Atlanta, Georgia; Emory University, Atlanta, GA

## Abstract

**Background:**

Large facilities handling dangerous pathogens often rely on complex and costly effluent decontamination systems to safely treat biohazardous liquid waste before discharging it into public sewer systems. However, such infrastructure is not practical for smaller facilities, including hospital-based biocontainment units caring for patients with high-consequence infectious diseases (HCIDs). To mitigate the potential risk associated with toileted waste from HCID patients, some units preemptively add disinfectant solutions to the toilet bowl before patient use. This study biologically validates the effectiveness of that practice.

**Methods:**

Three laboratory experiments were conducted to evaluate the ability of EPA-registered hospital-grade disinfectants to inactivate *Bacillus subtilis* spores—used as a surrogate for hard-to-kill pathogens—in matrices designed to simulate toilet bowl waste. The disinfectants tested were: Micro-Chem Plus (a dual quaternary ammonium compound), OxyCide (a combination of hydrogen peroxide and peracetic acid), and Altra (a dichlor-based disinfectant). The experiments assessed the disinfectants’ efficacy against (1) spores suspended in phosphate-buffered saline (PBS) (2) spores inoculated into stool and homogenized in PBS, simulating liquid stool, and (3) spores embedded in a sphere of stool and submersed in PBS, simulating solid stool in toilet water.

**Results:**

In the PBS control experiment, both OxyCide and Altra achieved effective inactivation of *B. subtilis* spores within a 5-minute contact time, whereas Micro-Chem Plus did not. In the liquid stool simulation, OxyCide and Altra again demonstrated effective inactivation after 5 minutes. However, neither disinfectant was effective in the solid stool simulation, indicating limited penetration or reduced efficacy in that context.

**Conclusion:**

Adding hospital-grade disinfectants to the toilet bowl before use by HCID patients may help reduce the risk of biohazardous material entering plumbing and municipal wastewater systems—specifically when the waste is liquid in consistency. However, this method appears ineffective for disinfecting solid stool, suggesting a need for alternative waste treatment strategies for such cases.

**Disclosures:**

Colleen S. Kraft, MD, MSc, Rebiotix Ferring: Advisor/Consultant|Vedanta: Advisor/Consultant

